# Family matters: skin microbiome reflects the social group and spatial proximity in wild zebra finches

**DOI:** 10.1186/s12898-020-00326-2

**Published:** 2020-11-13

**Authors:** Kathrin Engel, Helga Pankoke, Sebastian Jünemann, Hanja B. Brandl, Jan Sauer, Simon C. Griffith, Jörn Kalinowski, Barbara A. Caspers

**Affiliations:** 1 Department of Behavioural Ecology, Bielefeld University, Konsequenz 45, 33615 Bielefeld, Germany; 2 Evonik Nutrition & Care GmbH, Kantstr. 2, 33790 Halle, Germany; 3 Center for Biotechnology (CeBiTec), Bielefeld University, Sequenz 1, 33615 Bielefeld, Germany; 4 Faculty of Technology, Bielefeld University, Universitätsstraße 25, 33615 Bielefeld, Germany; 5 Institute of Zoology, Behavioural Biology, University of Hamburg, Martin-Luther-King Platz 3, 20146 Hamburg, Germany; 6 Department of Biological Sciences, Macquarie University, Sydney, NSW 2109 Australia

**Keywords:** Family-specific, Social group, Bacterial communities, Nestling, Avian olfaction, Olfactory communication, Relatedness, Social environment, Microbiota

## Abstract

**Background:**

So far, large numbers of studies investigating the microbiome have focused on gut microbiota and less have addressed the microbiome of the skin. Especially in avian taxa our understanding of the ecology and function of these bacteria remains incomplete. The involvement of skin bacteria in intra-specific communication has recently received attention, and has highlighted the need to understand what information is potentially being encoded in bacterial communities. Using next generation sequencing techniques, we characterised the skin microbiome of wild zebra finches, aiming to understand the impact of sex, age and group composition on skin bacteria communities. For this purpose, we sampled skin swabs from both sexes and two age classes (adults and nestlings) of 12 different zebra finch families and analysed the bacterial communities.

**Results:**

Using 16S rRNA sequencing we found no effect of age, sex and family on bacterial diversity (alpha diversity). However, when comparing the composition (beta diversity), we found that animals of social groups (families) harbour highly similar bacterial communities on their skin with respect to community composition. Within families, closely related individuals shared significantly more bacterial taxa than non-related animals. In addition, we found that age (adults vs. nestlings) affected bacterial composition. Finally, we found that spatial proximity of nest sites, and therefore individuals, correlated with the skin microbiota similarity.

**Conclusions:**

Birds harbour very diverse and complex bacterial assemblages on their skin. These bacterial communities are distinguishable and characteristic for intraspecific social groups. Our findings are indicative for a family-specific skin microbiome in wild zebra finches. Genetics and the (social) environment seem to be the influential factors shaping the complex bacterial communities. Bacterial communities associated with the skin have a potential to emit volatiles and therefore these communities may play a role in intraspecific social communication, e.g. via signalling social group membership.

## Background

While most studies investigating the microbiome of birds have focused on gut microbiota, e.g. [1], only a few have addressed the microbiome of the skin, such as the facial skin [2], brood patch [3, 4], neck region [5] and uropygial gland [3, 5, 6]. However, feathers and skin act as barriers between the bird and its environment and are thus particularly important body sites for investigation. Feathers have been shown to carry a substantial bacterial load [7]. Until now, most studies have focused on keratinolytic bacteria such as the feather degrading *Bacillus licheniformis* and investigated their impact on feather coloration and body condition [8, 9]. The skin is also densely populated with microbes [3–5], but we are still lacking knowledge about how the skin microbiota is usually composed and which functions these microbial communities fulfil. The transfer of host-specific information via chemical cues emitted from bacteria is one potential function [10]. For example, young zebra finches are able to discriminate kin and non-kin by scent alone [11] and young blue tit nestlings modulate their begging behaviour in response to the scent of conspecific nestlings [12]. In this last example the blue tit nestlings used as odour donors were 7 days old, an age where the preen gland is not functioning yet (Caspers pers. observation), indicating that the volatiles used to distinguish between familiar/related and unfamiliar/unrelated conspecifics cannot (solely) originate from the preen gland secretion. As volatiles emitted from bacteria carry great potential to act as cues (see e.g. the fermentation hypothesis of chemical recognition [13]) we are convinced that the skin microbiome, although largely overlooked, may be of specific interest. Consequently, we examined here the skin microbiome of wild zebra finches and used 16S rRNA gene sequencing to characterise the skin microbial communities of different zebra finch family members. Samples comprised females, males and their offspring, giving us the possibility to investigate whether age, sex and the social group (i.e. the family) affect the skin microbiota composition.

Age effects on the microbiota are well supported for the gut microbiome in birds [14], but it has yet to be demonstrated for the bacterial communities of the skin. Similar to most altricial songbirds, zebra finches hatch sparsely covered with down feathers and develop rapidly into adults with closed plumage. During their nestling phase, they are in direct skin contact with the nest environment and their siblings, whereas direct skin contact with their parents is probably rather limited, as zebra finches do not develop a pronounced brood patch. Therefore, we may expect that nestlings and adults differ in their bacterial communities to some extent. However, at the same time, offspring and parents share the same environment, i.e. nest, and therefore may be very similar in the composition of their skin microbiome. Thus, we do not have a clear prediction whether to expect age differences or not in microbial diversity and composition.

Another factor in shaping the skin microbiome may be the bird’s sex. It could be considered the most important genetic effect as it implies changes in the level of hormones, especially during the reproductive periods which could be influential on the skin microbiome. Some sources hint for gender-specific gut microbiota in broiler chicks, e.g. bacterial communities differ between male and female chicks’ ileum [15] and caeca [16]. Investigations on the skin microbiome are scarce, but for the sea bird *Oceanodroma leucorhoa*, the factor sex crucially impacts on the bacterial community variation [3]. However, Taylor and colleagues [17], could not find a difference between samples of the oral cavity from male and female birds of prey and in a previous study from our lab no effect of sex was detected in the skin microbiome of estrildid finches [5].

Lastly, we explored whether skin bacterial communities may be affected by the social groups (i.e. families). From mammals we know that social interactions shape the microbiomes of socially living species like hyenas [18] and meerkats [19]. In both species, bacterial communities from special skin glands, the anal pouches, are transferred within the social environment during scent marking and therefore transferred to animals of the same social group [18, 19]. Studies like these underpin the importance of conspecifics and the individual’s family for the colonization of the skin with microbiota, but also for the role of skin microbiota in olfactory communication. The nest environment, as well as the social environment, influences the bacterial community on the skin of various bird species (reviewed in [10]) and can therefore be considered to be drivers for diversity in the skin microbiomes of different families. Based on this we expected that animals living in social groups and sharing the same environment harboured more similar skin microbiomes compared to those living in more distant environments. Thus, we predict that the social group (family) affects the composition of the skin bacterial community, i.e. we predict members within a family to be more similar in their skin bacterial community than members between different families.

In summary, we analysed samples from parents and their offspring from a natural population of zebra finches in the Australian desert. With the results that we present here, we aim to contribute to a better understanding of the microbiota occurring on the skin, the factors potentially influencing it under natural conditions and therewith potential information that may be transferred via skin microbiota.

## Results

**Fig. 1:**
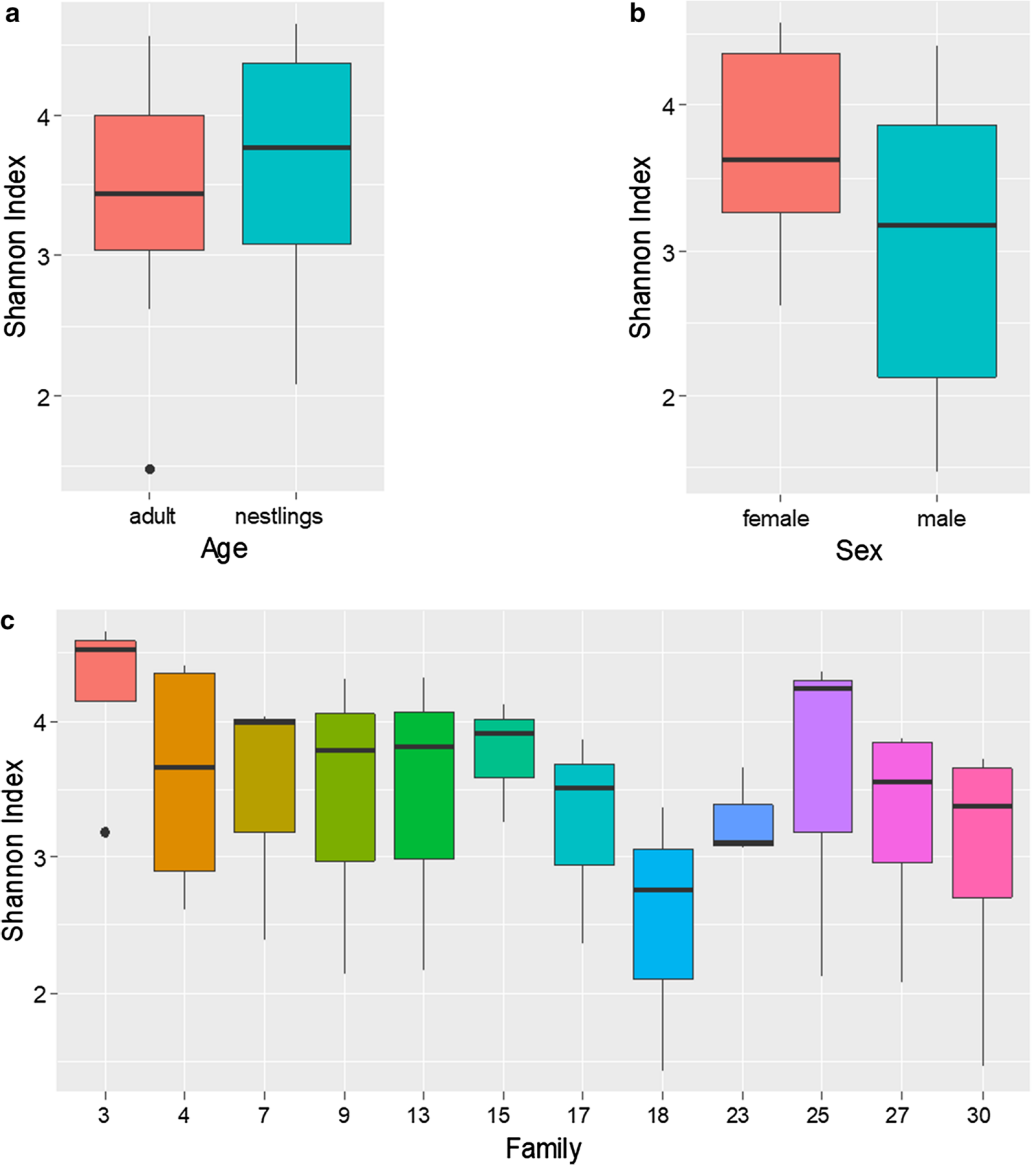
Comparison of Shannon diversity (alpha diversity) of the skin microbiota of wild living zebra finches. No effect of **a** bird age [*n* = 4 fully replicated families consisting of two adults (female, male) and two nestlings (offspring)], **b** sex (*n* = 5 families consisting of two adults and at least one nestling) and **c** family (*n* = 12 families with three to four individuals consisting of one or two adults and one or two nestlings) on Shannon diversity. X-axis labelling is representing the number of the families’ nest boxes. In the boxplots, the median is the bold horizontal line, the boxes refer to the interquartile range, and whiskers extend to max. 1.5 times the interquartile range, whereas dots are outliers

Using 16S rRNA gene sequencing, followed by bioinformatics processing, we detected a total of 1233 operational taxonomic units (OTUs) in 40 skin swab samples. In a first step we looked at alpha diversity, i.e. the species diversity of samples indicated by the Shannon diversity index. Bird age did not influence the bacterial diversity of the skin microbiota (sexually mature adults vs. nestlings) (Fig. 1a, ANOVA, *F*_1,14_ = 0.346, *P* = 0.566; *n* = 4 fully replicated families, in which both parents and two of their offspring were sampled). Further analyses showed that neither sex nor family affected bacterial diversity scores (Fig. 1b, sex: ANOVA, *F*_1,8_ = 1.078, *P* = 0.329; *n* = 5 families consisting of two adults (females vs. males) Fig. 1c, family: *F*_11,28_ = 0.715, *P* = 0.714, *n* = 12 families consisting of one or two adults and one or two nestlings, respectively).

**Fig. 2:**
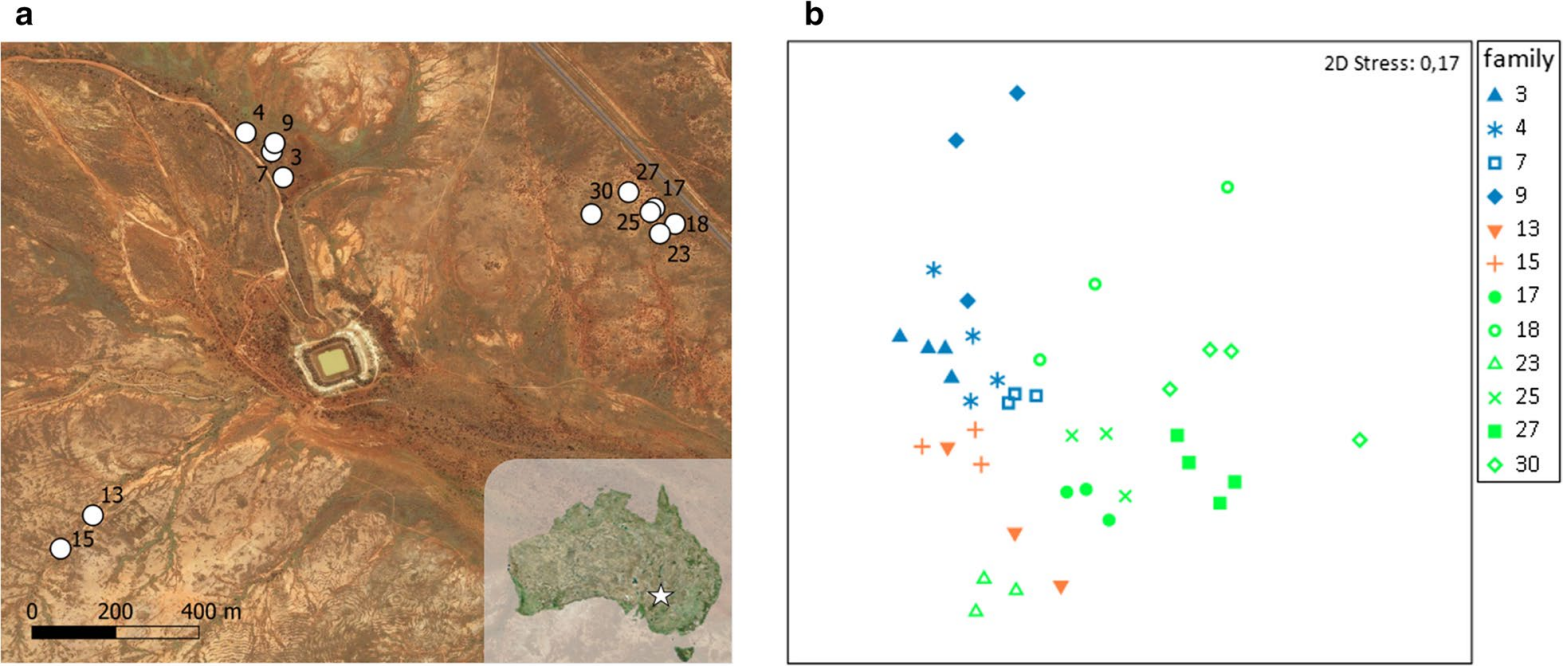
Impact of nest location on the skin microbiome. **a** Map of nest box locations (created with QGIS 3.8 [52]; both images are maps based on “Bing VirtualEarth”). Each circle indicates one nest box, marked with individual nest box (family) number. **b** Non-metric multidimensional ordination (nMDS) of skin microbiomes showing similarity between samples based on a Bray–Curtis similarity matrix (created with Primer-e [46]) The closer symbols appear on the plot, the more similar they are concerning their bacterial community. Blue-coloured shapes belong to samples from nest boxes 3, 4, 7 and 9 in the north, orange-coloured shapes to 13 and 15 in the west and green-coloured shapes to 17–30 in north-east. Symbols of same shape represent different members of a family (nest box). Axes of the nMDS plot are arbitrary and dimensionless

In a second step we analysed the beta diversity, i.e. the similarity between samples based on the Bray–Curtis similarity index, and found that the family significantly affected the composition of the bacterial skin community (one-way ANOSIM (factor *family*), R = 0.785, *P* = 0.001, Fig. 2b, Additional file 1). The microbial composition among family members was, on average, more similar than between members of different families. When focusing on the four families consisting of 2 adults and 2 nestlings, we found a significant effect of age (i.e. adult vs*.* nestling) (two-way nested ANOSIM (*age* nested in *family*), R = 0.625, *P* = 0.012). Finally, when looking at adult birds only, the sex of an individual did not affect the composition of the bacterial skin communities (ANOSIM, R = − 0.046, *P* = 0.741).

**Fig. 3:**
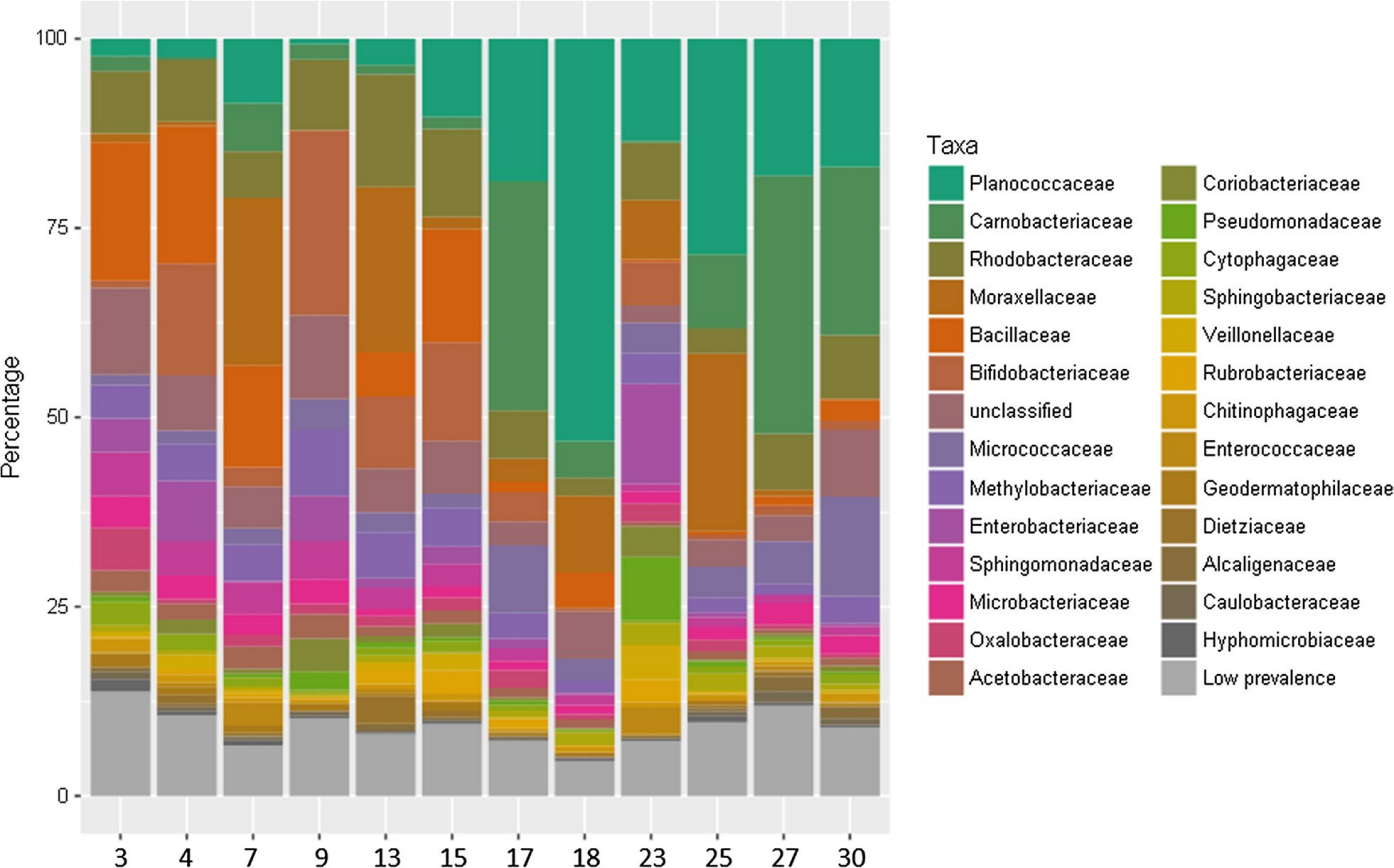
Taxonomic profiling of the skin microbiota of the zebra finch. The stack bar shows the most abundant families of the bacterial community based on 1233 OTUs using rarefied data (5536 read counts per sample). Low prevalence corresponds to ≤ 0.5% of the respective taxa relative to the absolute number of read counts. The order of the taxa in the legend reflects the relative average abundance of the respective taxa over all families. *n* = 12 families with three to four individuals consisting of one or two adults and one or two nestlings

Further taxonomic profiling of the zebra finch microbiome on a family level revealed that zebra finch families mainly shared the same taxa, yet differed in their respective abundance profiles (Fig. 3). In total, 142 bacterial families could be identified, of which 115 families were present with a prevalence of less than 0.5%. The remaining 27 bacterial families are illustrated in Fig. 3. The most abundant taxa over all samples were from the families *Planococcaceae*, followed by *Carnobacteriaceae*, *Rhodobacteraceae*, *Moraxellaceae* and *Bacillaceae* and were present in all 12 families but with differences in their abundance. Extremes ranged from about 50% *Planococcaceae* in the samples of family 18 to a few per cent in family 9 (Fig. 3).

**Fig. 4:**
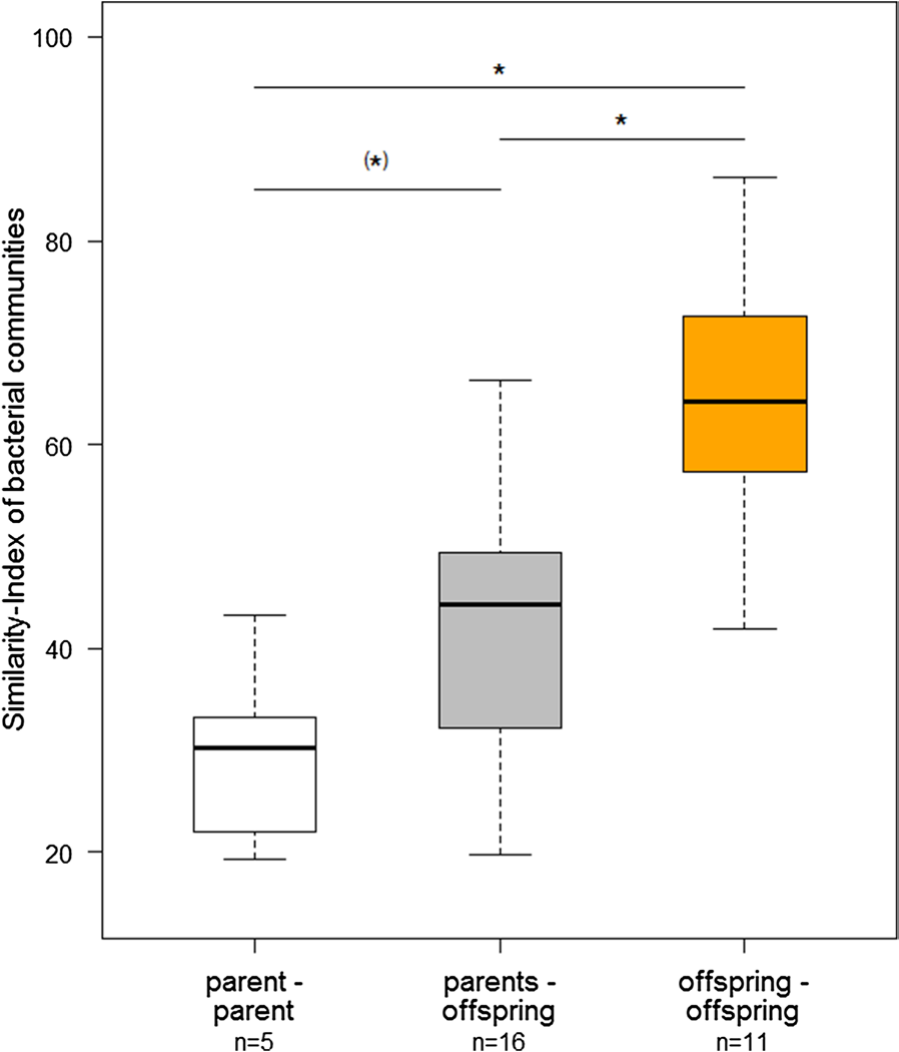
Comparison of the similarity of bacterial communities on the skin between different family members (beta diversity). The parent-parent group comprises all pairs (male and female) that bred and raised offspring together (*n* = 5). The parents-offspring group includes all parents (either male or female) and their offspring, respectively (*n* = 16). The offspring-offspring group comprises all sibling pairs (*n* = 11). Asterisks indicate significant difference between groups (*P* < 0.05, post-hoc Wilcoxon rank sum tests). Similarities are based on Bray–Curtis similarity index. In the boxplots, the median is the bold horizontal line, the boxes refer to the interquartile range, and whiskers extend to max. 1.5 times the interquartile range

To better understand the impact of (social) environment and relatedness, we compared the pairwise similarity of the microbial communities among the different family member pairs (parent-parent, offspring-offspring and offspring-parent). Within families, we found that the similarity of the bacterial composition differed significantly among these groups (Fig. 4, Kruskal–Wallis rank sum test, Chi^2^ = 14.815, df = 2, *P* = 0.0006). Post-hoc pairwise comparisons via Wilcoxon rank sum tests revealed that within families, the offspring (*n* = 11) shared the highest similarity in microbiome composition, whereas the bacterial composition of the skin was less similar for parents and their offspring (*n* = 16) and least similar for parent-parent pairs (*n* = 5). The similarity between siblings differed significantly from both the similarity between paired adults (male and female from one family; Fig. 4, offspring-offspring vs. parent-parent *P* = 0.0037) and the similarity between adults and their offspring (Fig. 4, offspring-offspring vs. parents-offspring *P* = 0.0029). The similarity between parents, however, did only marginally differ from the similarity between adults and their offspring (Fig. 4, parent-parent vs. parents-offspring *P* = 0.0616). Mother and father did not differ in their similarity to their offspring (Wilcoxon rank sum test, W = 51, *P* = 0.1672) and were therefore addressed as “parents”.

In addition, we analysed the influence of nest box locality as a spatial/environmental factor on the skin microbiome. We found that spatial coordinates significantly correlated with the skin microbiome (Fig. 2, Mantel test, R = 0.163, *P* = 0.019), indicating an environmental or spatial impact on the zebra finch microbiome.

## Discussion

Environmental factors as well as host genetics are assumed to be the main factors shaping microbial communities of the host. However, the impact of these factors on avian skin microbiota is largely unknown. Studying wild zebra finches, we revealed an impact of environment (here spatial, i.e. distance between nest boxes, as well as the social environment) and potentially host genetics on the skin microbiome in this highly social bird species.

### No influence of sex, but age on skin bacteria

As expected, we did not find an effect of sex, neither on the skin bacterial community diversity (Fig. 1b) nor on its composition in the wild living zebra finches. This result contrasts with the outcome of a study by Pearce and colleagues on the skin microbiome of wild Leach’s storm petrels [3], but is in line with former findings in zebra finches housed under laboratory conditions [5]. The influence of sex may therefore vary between different bird species and further investigations would be helpful to make a sound statement.

While we found no evidence for an impact of age on the skin microbiota diversity in our study population (Fig. 1a) we found a significant difference between the composition of nestling and adult skin bacteria. Although we had no clear prediction, this highlights an impact of the habitat, i.e. the nest environment. The skin of adult zebra finches is covered with feathers and may not be substantially influenced by the environment, whereas the skin of the nestlings was barely covered by down feathers at the time of our sampling. Nestlings usually defecate on the nest fringe without parents removing the droppings. As nestling and adult skin microbiota differ in composition, it seems plausible that nestlings’ skin bacteria are to a certain extent shaped by the nest environment, e.g. the faeces, at first and fledglings only later develop a stable “adult microbiome” such as samples from microbiota of the digestive tract suggest [15, 17, 20]. In humans, the birthing method has been shown to be an important factor in shaping an infant’s skin microbiome [21]. It is either shaped by their mother’s vaginal flora (vaginal birth) or the mothers’ skin and the environment (caesarean section) [22]. In birds, however, bacterial communities on eggshells seem to derive mainly from surrounding nest material and are also horizontally transmitted from the skin and feathers of the breeding adults, rather than from the female’s cloaca [23]. Although we found a significant effect of age on skin bacterial communities, it is important to mention that individuals within a family are overall more similar to each other than individuals of different families.

### Family-specific skin microbiome and spatial effects

While our data did not reveal an impact of family on the alpha diversity of skin microbial communities (Fig. 1c), we found a strong signature of family on the composition of the skin microbiome (Figs. 2b, 3). Thus, on average the microbial composition among family members was more similar than between families (Figs. 2b). These differences emerged especially because families differed in the relative abundance of bacterial species, but not so much in the presence/absence of specific bacteria (Fig. 3).

Further, the spatial proximity of the nesting locations was reflected in the bacterial community compositions (Fig. 2). The zebra finches in our study population bred in a large colony extending over 1.5 kms, consisting of several smaller clusters of nest boxes in higher densities (distance between neighbouring boxes within clusters = 10.4 ± 4.8 SD meters). Zebra finches share strong social relationships with their spatially close neighbours that involve different social interactions, such as visiting each other’s nests [24], actively synchronising their reproduction [25], and foraging together [26]. These strong social bonds between individuals can be maintained over multiple seasons and persist even outside of reproductive periods [26]. The prolonged social interactions give some opportunity for direct exchange of bacteria, but shared foraging spaces and resting places also mean that individuals are in contact with a similar environmental bacterial pool. This circumstance may explain the high number of shared taxa between the families inhabiting nest boxes in close proximity but warrants further investigation.

Literature shows that environment and spatial proximity have a major impact on the gut microbiome of birds [27, 28] and even on the microbiome on feathers and in the uropygial gland [6]. Frequent social interactions can lead to an exchange of bacteria from one animal to the other. For example, frequent social interactions lead to a homogeneity among the gut microbial communities within and between generations in chimpanzees [29]. In socially living carnivores like hyenas [18] and meerkats [19], bacterial communities are shared by individuals of the same social group. Such a transfer of bacteria has not been shown for skin bacteria in particular, but Kulkarni and Heeb [30] demonstrated that bacteria applied on zebra finch plumage can be transmitted to the gut of co-housed conspecifics, presumably via allo-preening.

To explore a potential effect of genetic similarity on skin bacterial communities, we compared similarities of the skin microbiome among different family member groups (Fig. 4). The high offspring-offspring similarity among sibling pairs, compared to the lower offspring-parent similarity could be indicative for a strong influence of the environment. This is not very surprising when we consider that the nestlings sat together in a nest with very close body contact at the age they were sampled. Both, parents and their offspring, as well as the siblings most probably have the same degree of kinship (i.e. 0.5). However, differences in the similarity of skin microbiomes especially between the group of paired adults and the offspring group, are also indicative for a genetic influence on the microbiome of a bird’s skin. The paired adults are unlikely to be closely related to each other, whereas offspring in the same nest are typically always full siblings [31]. The high similarity in the skin microbiome of siblings may therefore also reflect the birds’ relatedness. As we do not have paternity data for this study, this warrants future investigations. However, genetic components on the bacterial community composition in birds have been shown in former studies, demonstrating that the genetic background of chickens is highly influential [32]. Evidence for the same pattern in skin bacteria is provided by a study in estrildid finches. They harboured individual-specific bacteria on their skin and differed in their skin microbiome despite experiencing the same environmental and dietary conditions [5]. We conclude that the bacterial community of zebra finch skin is, in part, modulated by the host and therefore leads to differences in bacterial community structure.

### Missing links and further implications

We show in our study that in wild zebra finches, spatial proximity, the environment and social interactions impact on the bacterial communities on the skin of family members, together with a potential influence of host genotype. To further investigate the origin of bacterial communities on the skin of birds and to disentangle environmental from genetic effects, further studies separating the different factors in a standardised environment may be promising.

Currently, we do not know much about the potential function of skin bacteria in birds. The finding that the microbial communities encode potentially fitness relevant information about the host, such as group membership, social environment and genetic similarity [6, 18] allows us to speculate that bacteria are involved in signalling this information during intraspecific communication. Although the exact pathways are not fully understood, one idea is that bacteria produce volatiles used in olfactory communication [13]. Although our study was not designed to prove an impact of bacteria on olfactory communication, the fact that birds and in particular zebra finches are known to use odours during intraspecific communication [11, 33, 34], in conjunction with the recent finding that bacteria are involved in odour production in the dark eyed junco [35], support this hypothesis.

## Conclusions

Using high-throughput sequencing, we found that wild zebra finch family members harbour highly similar bacterial communities on their skin with respect to community composition and relative abundance, thus indicating a family-specific skin microbiome. The similarity in the skin microbiome of families in close spatial proximity provides an indication for the influence of the (social) environment on the skin bacterial communities of these birds. Furthermore, we found that closely related individuals (i.e. siblings sharing the same nest and a parental unit and its offspring) share significantly more bacterial taxa than non-related animals, which is consistent with a genetic component shaping the skin microbiome of this species of estrildid finches, although the contribution of the intimately shared environment needs to be accounted for. Based on these findings, we recently performed a follow-up study using a cross-fostering approach that will enable us to set environmental factors apart from genetic factors in captive zebra finches.

Our study shows that skin bacteria have the potential to provide important, fitness relevant information about the host. Although we can currently only speculate about the exact pathways, bacteria may play an important role in intraspecific communication, for example through volatiles produced during bacterial fermentation.

## Methods

### Study organism and bacterial sampling

In order to investigate the skin microbiota of wild zebra finches (*Taeniopygia guttata*), individuals from a breeding colony around the UNSW Arid Zone Research Station at Fowlers Gap, Australia (31° 05′ 13.1ʺ S 141° 42′17.4ʺ E), details, see: [25, 36], were sampled during September and October 2016. Study animals comprised twelve zebra finch families, consisting of three to four individuals (*n* = 40; 9 females, 8 males, 23 nestlings). Families were defined as two parents and two nestlings (family of four individuals, *n* = 4) or two parents and one nestling (family of three individuals, *n* = 1) or one parent with two nestlings (family of three individuals, *n* = 7). Samples that did not fit in one of these groups were excluded. Parents were always a male and a female of unknown age, the offspring was between six and ten days old at the day of sampling. Offspring sex was not identifiable at this developmental stage.

For the sampling, adults were caught with nest box traps. When a bird was caught, it was retrieved from the nest box and sampled immediately, wearing a fresh pair of sterile gloves for every bird. Adults were then held in bird bags until catching was completed and afterwards the chicks were sampled, taking each out of the nest and sampling it separately, wearing a fresh pair of gloves. All birds were sampled with a nylon flocked swab (ESwab, Copan Italia, Italy) on the bare skin parts around the preen gland. In order to do so, a swab was stroked on the skin adjacent to the preen gland, sparing the skin of the gland itself to avoid taking up preen gland secretions (for a detailed description see [5]). As our aim was to sample skin bacteria, the feathers of adult birds were parted and the swab rubbed on the skin, trying to avoid feather contact. Nevertheless, touching the down feathers could not be avoided completely. Samples from every family were taken at the same day, whenever possible. Swabs were transferred to liquid Amies medium immediately and transported on ice in a cooler box until storage at − 20 °C within 5 h.

### DNA extraction

Shipping of the frozen samples occurred in February 2017 on dry ice from Australia. In Germany, DNA of all samples was extracted within one week after arrival using the BiOstic Bacteremia DNA Isolation Kit (MO BIO Laboratories, Carlsbad, CA, USA) according to the manufacturer's instructions with minor adjustments. The entire volume of 800 µl liquid Amies medium containing floating bacteria was used (instead of a sample of 1800 µl blood as instructed in the kit’s manual). Furthermore, samples in bead tubes were shaken vertically for 10 min at 50 Hz in a TissueLyser instrument (TissueLyser LT, Qiagen, Venlo, Netherlands).

### Library preparation and DNA sequencing

Library preparation was performed following the Illumina 16s Metagenomic Library Prep Guide, 15044223-b and the detailed description provided by Jervis-Bardy et al. [37]. We used a slightly modified protocol to accommodate our need for low DNA concentrations. The primer pair we used in our study is derived from Klindworth et al. [38] and targeted the hypervariable regions V3 and V4 of the 16S rRNA gene (S-D-Bact-0341-b-S-17: 5′-CCTACGGGNGGCWGCAG-3′ and S-D-Bact-0785-a-A-21: 5′-GACTACHVGGGTATCTAATCC-3′). Primers were connected to Illumina overhang adapter sequences as follows:

forward: 5′-TCGTCGGCAGCGTCAGATGTGTATAAGAGACAG-3′,

reverse: 5′-GTCTCGTGGGCTCGGAGATGTGTATAAGAGACAG-3′.

For a detailed description see Supporting information S1 in [5].

The libraries were sequenced on an Illumina MiSeq system (Illumina, Inc., San Diego, CA, USA) in paired-end mode (2 × 300 sequencing cycles) at the CeBiTec, Bielefeld University, using the MiSeq Reagent Kit v3 (Illumina, Inc., San Diego, CA, USA) following the manufacturer’s manual.

### Bioinformatic data processing

Data was processed analogous to our previous study [5], with some minor adjustments, as described briefly in the following. MiSeq paired-end reads were assembled with Flash v1.2.11 [39] in an iterative manner coupled with read clipping based on average quality score values using sickle v1.33 [40] in order to achieve a maximum assembly rate. Reads initially failing the read assembly were clipped to a q20 quality threshold and tried to assemble again. This process was repeated with a repeatedly increased quality threshold by 3 up to the limit of q35. Assembled PE reads were then screened for matching forward and reverse amplification primers, in both directions consecutively, using cutadapt v2.1 [41], and matched primers were trimmed during this procedure. Reads matching the primers in reverse direction were reverse complemented hereby. Then, mothur v.1.41.3 [42] was used to de-replicate, align, filter, and de-noise reads in accordance to the mothur MiSeq standard operating procedure. Read sets were consecutively screened for chimeras and clustered into OTUs with a percent identity threshold of 97% using the USEARCH v8.0.147 tool suite [43]. Finally, each OTU was taxonomically labelled based on naïve Bayesian read classification in mothur using the full SILVA database v132 [44] as a reference and a confidence cut-off of 80%. Please see Engel et al. [5] for more details on the bioinformatics processing, invoked commands and used parameters.

### Data filtering and statistical analyses

All statistical analyses were performed using R version 3.3.3. [45] and Primer-e software version 7 [46]. After bioinformatic processing, we applied additional filtering steps to the OTU table as described in Pankoke et al. [47]. In short, only bacterial OTUs that could be classified at phylum level were used for further filtering steps. OTUs with less than 10 read counts per cluster as well as OTUs that did not occur in at least two samples were removed from the dataset. After filtering, 1378 OTUs with a mean read count of 24,194 ± 10,885 were used for a rarefaction analysis. Samples were rarefied to 5536 read counts, using *rrarefy()* from the *vegan* R package [48] resulted in 1233 OTUs.

Using the respective function implemented in *vegan*, we estimated bacterial diversity by calculating the Shannon diversity index (alpha diversity). To overcome limitations of unbalanced sampling, i.e. not always two parents and two siblings per family, we compared diversity scores with linear models on reduced datasets. To estimate the impact of bird age on microbial diversity, we selected the fully replicated families consisting of two adults (female, male) and two nestlings (offspring) (*n* = 4). To estimate the influence of sex on microbial diversity, we analysed the paired adults from those *n* = 5 families that consisted of two adults and at least one nestling. And to estimate the influence of family on diversity scores, we analysed all *n* = 12 families that consisted of three to four individuals (one or two adults and one or two nestlings). Residuals of the models were visually inspected for normality and deviation from homoscedasticity [49].

The rarefied OTU abundance data were transformed (log10(x + 1)) prior to statistical analysis. For all further analyses investigating beta diversity, we computed a pairwise similarity matrix based on the Bray–Curtis similarity index and analysed potential differences between a priori defined groups (here: age, sex and families) using a non-parametric analysis of similarities (ANOSIM). We performed the ANOSIM using Primer 7 [46]. To visualise the similarities or differences in microbial communities, we used a non-metric multidimensional scaling plot (nMDS).

Moreover, we explored whether the amount of shared skin bacterial communities differed between members within a family. Therefore, we calculated pairwise similarity indices (based on Bray–Curtis similarity) for the following pairs: parent-parent, parent–offspring and offspring-offspring using the rarefied OTU table (using Primer 7). To test whether similarities differed among these groups we used a Kruskal–Wallis test, followed by post-hoc Wilcoxon rank sum tests for comparisons between groups (using R).

To test the hypothesis that spatial or environmental factors influenced the skin microbiome of the zebra finch, the rarefied OTU table was first converted to a distance matrix using the Bray–Curtis distance. Next, we calculated a distance matrix of the spatial coordinates of the nest boxes using *distm(coordinates (), fun* = *distHaversine)* from the packages *geosphere* [50] and *sp* [51]. Finally, we performed a Mantel test as implemented in *vegan* to analyse whether the distance matrix of the rarefied OTU table and the distance matrix of the spatial coordinates were correlated. The matrices used for the analyses are attached as Additional file 2.

Taxonomic profiling of the skin microbiota of the zebra finch was done according to Pankoke et al. [47]. In short, read counts of all OTUs belonging to one specific taxon were summed up and normalized by the total number of read counts, yielding proportions. Each taxonomic level was then deduced from the normalized OTU counts by summing up the taxonomic classifications on different levels. The respective proportions of the different taxa were ranked by decreasing value and plotted as stack bars. All taxa with less than 0.5% of prevalence were summed up and were being classified as of “low prevalence”.

## Supplementary information

**Additional file 1:** Supplementary figures.

**Additional file 2:** Excel file containing metadata, OTUs and sequences used for analyses in R and Primer-e.

## Data Availability

The dataset generated during the current study is available in the ENA repository under the project number PRJEB37771 (https://www.ebi.ac.uk/ena/data/view/PRJEB37771). The OTU table is attached as an additional file to this manuscript.

## Abbreviations

ANOSIM: Analysis of similarity
ANOVA: Analysis of variance
OTU: Operational taxonomic unit

## References

[1] Waite DW, Taylor MW (2015). Exploring the avian gut microbiota: current trends and future directions. Front Microbiol.

[2] Roggenbuck M, Bærholm Schnell I, Blom N, Bælum J, Bertelsen MF, Sicheritz-Pontén T (2014). The microbiome of New World vultures. Nat Commun.

[3] Pearce DS, Hoover BA, Jennings S, Nevitt GA, Docherty KM (2017). Morphological and genetic factors shape the microbiome of a seabird species (*Oceanodroma leucorhoa*) more than environmental and social factors. Microbiome.

[4] van Veelen HPJ, Falcao Salles J, Tieleman BI (2017). Multi-level comparisons of cloacal, skin, feather and nest-associated microbiota suggest considerable influence of horizontal acquisition on the microbiota assembly of sympatric woodlarks and skylarks. Microbiome.

[5] Engel K, Sauer J, Jünemann S, Winkler A, Wibberg D, Kalinowski J (2018). Individual- and species-specific skin microbiomes in three different estrildid finch species revealed by 16S amplicon sequencing. Microb Ecol.

[6] Whittaker DJ, Gerlach NM, Slowinski SP, Corcoran KP, Winters AD, Soini HA (2016). Social environment has a primary influence on the microbial and odor profiles of a chemically signaling songbird. Front Ecol Evol.

[7] Dille JW, Rogers CM, Schneegurt MA (2016). Isolation and characterization of bacteria from the feathers of wild Dark-eyed Juncos (*Junco hyemalis*). Auk.

[8] Gunderson AR, Forsyth MH, Swaddle JP (2009). Evidence that plumage bacteria influence feather coloration and body condition of eastern bluebirds *Sialia sialis*. J Avian Biol..

[9] Leclaire S, Pierret P, Chatelain M, Gasparini J (2014). Feather bacterial load affects plumage condition, iridescent color, and investment in preening in pigeons. Behav Ecol.

[10] Maraci Ö, Engel K, Caspers BA (2018). Olfactory communication via microbiota: what is known in birds?. Genes.

[11] Caspers BA, Hagelin JC, Paul M, Bock S, Willeke S, Krause ET (2017). Zebra finch chicks recognise parental scent, and retain chemosensory knowledge of their genetic mother, even after egg cross-fostering. Sci Rep.

[12] Rossi M, Marfull R, Golüke S, Komdeur J, Korsten P, Caspers BA (2017). Begging blue tit nestlings discriminate between the odour of familiar and unfamiliar conspecifics. Funct Ecol.

[13] Albone ES, Perry GC (1976). Anal sac secretion of the red fox, *Vulpes vulpes*; volatile fatty acids and diamines: Implications for a fermentation hypothesis of chemical recognition. J Chem Ecol.

[14] Barbosa A, Balagué V, Valera F, Martínez A, Benzal J, Motas M (2016). Age-related differences in the gastrointestinal microbiota of chinstrap penguins (*Pygoscelis antarctica*). PLoS ONE.

[15] Lumpkins BS, Batal AB, Lee M (2008). The effect of gender on the bacterial community in the gastrointestinal tract of broilers. Poult Sci.

[16] Lee K-C, Kil DY, Sul WJ (2017). Cecal microbiome divergence of broiler chickens by sex and body weight. J Microbiol.

[17] Taylor MJ, Mannan RW, U’Ren JM, Garber NP, Gallery RE, Arnold AE (2019). Age-related variation in the oral microbiome of urban Cooper’s hawks (*Accipiter cooperii*). BMC Microbiol.

[18] Theis KR, Schmidt TM, Holekamp KE (2012). Evidence for a bacterial mechanism for group-specific social odors among hyenas. Sci Rep.

[19] Leclaire S, Jacob S, Greene LK, Dubay GR, Drea CM (2017). Social odours covary with bacterial community in the anal secretions of wild meerkats. Sci Rep.

[20] Kohl KD, Brun A, Caviedes-Vidal E, Karasov WH (2019). Age-related changes in the gut microbiota of wild house sparrow nestlings. Ibis.

[21] Funkhouser LJ, Bordenstein SR (2013). Mom knows best: the universality of maternal microbial transmission. PLoS Biol.

[22] Domínguez-Bello MG, Costello EK, Contreras M, Magris M, Hidalgo G, Fierer N, Knight R (2010). Delivery mode shapes the acquisition and structure of the initial microbiota across multiple body habitats in newborns. Proc Natl Acad Sci USA.

[23] van Veelen HPJ, Salles JF, Tieleman BI (2018). Microbiome assembly of avian eggshells and their potential as transgenerational carriers of maternal microbiota. ISME J.

[24] Brandl HB, Griffith SC, Laaksonen T, Schuett W (2019). Begging calls provide social cues for prospecting conspecifics in the wild zebra finch (*Taeniopygia guttata*). Auk.

[25] Brandl HB, Griffith SC, Schuett W (2019). Wild zebra finches choose neighbours for synchronized breeding. Anim Behav.

[26] Brandl HB, Griffith SC, Farine DR, Schuett W (2019). Wild zebra finches that nest synchronously have long-term stable social ties. J Anim Ecol.

[27] Hird SM, Carstens BC, Cardiff SW, Dittmann DL, Brumfield RT (2014). Sampling locality is more detectable than taxonomy or ecology in the gut microbiota of the brood-parasitic Brown-headed Cowbird (*Molothrus ater*). PeerJ.

[28] Gillingham MAF, Béchet A, Cézilly F, Wilhelm K, Rendón-Martos M, Borghesi F (2019). Offspring microbiomes differ across breeding sites in a panmictic species. Front Microbiol.

[29] Moeller AH, Foerster S, Wilson ML, Pusey AE, Hahn BH, Ochman H (2016). Social behavior shapes the chimpanzee pan-microbiome. Sci Adv.

[30] Kulkarni S, Heeb P (2007). Social and sexual behaviours aid transmission of bacteria in birds. Behav Process.

[31] Griffith SC, Holleley CE, Mariette MM, Pryke SR, Svedin N (2010). Low level of extrapair parentage in wild zebra finches. Anim Behav.

[32] Zhao L, Wang G, Siegel P, He C, Wang H, Zhao W (2013). Quantitative genetic background of the host influences gut microbiomes in chickens. Sci Rep.

[33] Golüke S, Bischof H-J, Engelmann J, Caspers BA, Mayer U (2019). Social odour activates the hippocampal formation in zebra finches (*Taeniopygia guttata*). Behav Brain Res.

[34] Krause ET, Caspers BA (2018). Do diamond firetails (*Stagonopleura guttata*) recognise the scent of their nest as other estrildid finches do?. Emu.

[35] Whittaker DJ, Slowinski SP, Greenberg JM, Alian O, Winters AD, Ahmad MM (2019). Experimental evidence that symbiotic bacteria produce chemical cues in a songbird. J Exp Biol..

[36] Griffith SC, Pryke SR, Mariette M (2008). Use of nest-boxes by the zebra finch (*Taeniopygia guttata*): implications for reproductive success and research. Emu.

[37] Jervis-Bardy J, Leong LEX, Marri S, Smith RJ, Choo JM, Smith-Vaughan HC (2015). Deriving accurate microbiota profiles from human samples with low bacterial content through post-sequencing processing of Illumina MiSeq data. Microbiome.

[38] Klindworth A, Pruesse E, Schweer T, Peplies J, Quast C, Horn M, Glöckner FO (2013). Evaluation of general 16S ribosomal RNA gene PCR primers for classical and next-generation sequencing-based diversity studies. Nucleic Acids Res.

[39] Magoč T, Salzberg SL (2011). FLASH: fast length adjustment of short reads to improve genome assemblies. Bioinformatics.

[40] Joshi NA, Fass JN. Sickle: a sliding-window, adaptive, quality-based trimming tool for FastQ files (Version 1.33) [Software]. 2011. https://github.com/najoshi/sickle.

[41] Martin M (2011). Cutadapt removes adapter sequences from high-throughput sequencing reads. EMBnet J.

[42] Schloss PD, Westcott SL, Ryabin T, Hall JR, Hartmann M, Hollister EB (2009). Introducing mothur: open-source, platform-independent, community-supported software for describing and comparing microbial communities. Appl Environ Microbiol.

[43] Edgar RC (2010). Search and clustering orders of magnitude faster than BLAST. Bioinformatics.

[44] Quast C, Pruesse E, Yilmaz P, Gerken J, Schweer T, Yarza P (2013). The SILVA ribosomal RNA gene database project: improved data processing and web-based tools. Nucleic Acids Res.

[45] R Core Team. R: a language and environment for statistical computing. Vienna: R Foundation for Statistical Computing; 2017. https://www.R-project.org/.

[46] Clarke KR, Gorley RN, Somerfield PJ, Warwick RM (2014). Change in marine communities: an approach to statistical analysis and interpretation.

[47] Pankoke H, Maus I, Loh G, Hüser A, Seifert J, Tilker A (2020). F5Evaluation of commercially available DNA extraction kits for the analysis of the broiler chicken cecal microbiota. FEMS Microbiol Lett..

[48] Oksanen J, Blanchet FG, Kindt R, Legendre P, Minchin PR, O’hara RB, et al. vegan: community ecology package. R package. 2017.

[49] Zuur AF, Ieno EN, Elphick CS (2010). A protocol for data exploration to avoid common statistical problems. Methods Ecol Evol.

[50] Hijmans RJ, Williams E, Vennes C (2017). geosphere: spherical trigonometry. R package. Spher Trigon.

[51] Pebesma EJ, Bivand RS. sp: classes and methods for spatial data. R package. 2005.

[52] QGIS Development Team. QGIS Geographic Information System. Open Source Geospatial Foundation Project; 2019. https://qgis.osgeo.org.

