# Supplementary material for: Family matters: skin microbiome reflects the social group and spatial proximity in wild zebra finches
Source: BMC Ecol. 2020 Nov 13;20:58. doi: 10.1186/s12898-020-00326-2 (PMC7664024; doi:10.1186/s12898-020-00326-2)

Three nMDS plots showing the families from Fig. 2 B, separated into the geographical clusters and visually illustrating the different sexes and therefore age classes.


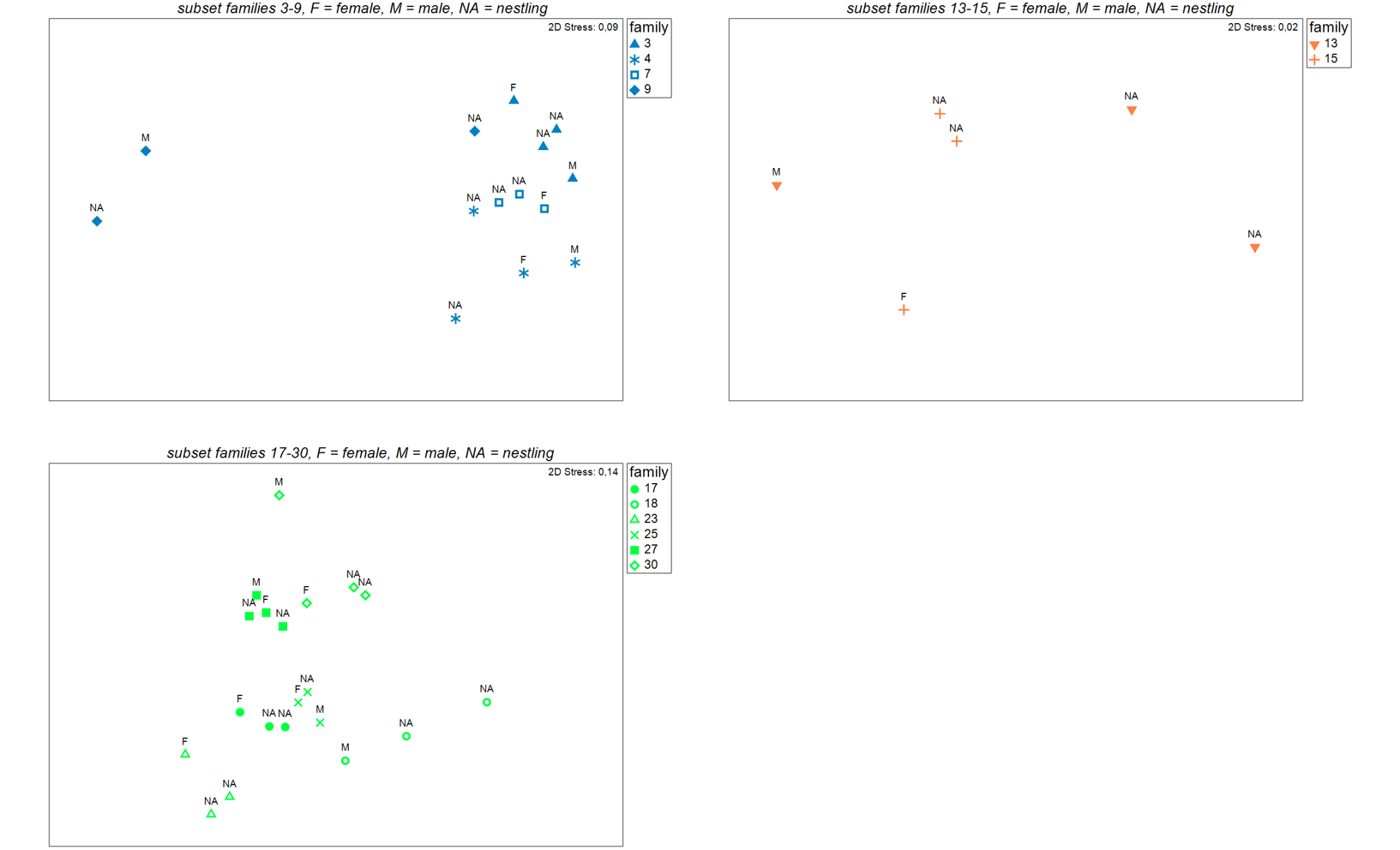

Supplement: Supplementary file 1 — Additional file 1: Supplementary figures. [file 12898_2020_326_MOESM1_ESM.docx]
